# Chauncey S. Bigelow

**Published:** 1915-01

**Authors:** 


CHAUNCEY S. BIGELOW.—Mr. C. S. Bigelow, the President of the Ransom & Randolph Co., of Toledo, publishers of the *Dental Summary*, died at his home in Toledo December 2, 1914, of angina pectoris and myocarditis, induced by inhaling accidentally acetylene gas from an automobile cylinder. It seems unreal that a man of so much vigor, good health, and occupying so important a place of usefulness in life's work can be so suddenly taken from his sphere of usefulness. Mr. Bigelow was in the prime of life and was making a great name and reputation for his business concern. He was 52 years of age, and had attained his position as head of this great business house, starting as a clerk, in a very short period of time.

The secret of his success may be accounted for primarily because of his tireless energy and complete devotion to this particular business. As a clerk and salesman he always worked and always planned to succeed in whatever he undertook, and he did. He was conscientious, friendly, and had great executive capacity, and these characteristics, with hard work and natural ability to discern the tendency of the profession development, enabled him to give his house the standing which it has today in the business world. He had a refined taste for things other than business. In his home life he was very happy; he was blessed with a wife and three children, who were his greatest inspiration and for whom he was always ready to make any necessary personal sacrifice. He was also devoted to his business associates and had a deep personal attachment for Mr. Crandall who developed in the business along with him. His death is a great loss to his family and business associates, as well as to the profession. He has passed on from his work and friends, but his personal contact will always remain a precious memory and help to soften our attitude towards those tasks in life which seem hard and impossible to us, inspiring us with new hopes and greater ideals.

